# Common polygenic risk for autism spectrum disorder (ASD) is associated with cognitive ability in the general population

**DOI:** 10.1038/mp.2015.12

**Published:** 2015-03-10

**Authors:** T-K Clarke, M K Lupton, A M Fernandez-Pujals, J Starr, G Davies, S Cox, A Pattie, D C Liewald, L S Hall, D J MacIntyre, B H Smith, L J Hocking, S Padmanabhan, P A Thomson, C Hayward, N K Hansell, G W Montgomery, S E Medland, N G Martin, M J Wright, D J Porteous, I J Deary, A M McIntosh

**Affiliations:** 1 Division of Psychiatry, University of Edinburgh, Edinburgh, UK; 2 QIMR Berghofer Medical Research Institute, Brisbane, QLD, Australia; 3 Centre for Cognitive Ageing and Cognitive Epidemiology, University of Edinburgh, Edinburgh, UK; 4 Division of Applied Health Sciences, University of Aberdeen, Aberdeen, UK; 5 Institute of Cardiovascular and Medical Sciences, University of Glasgow, Glasgow, UK; 6 Medical Genetics Section, Molecular Medicine Centre, Institute of Genetics and Molecular Medicine, University of Edinburgh, Edinburgh, UK; 7 MRC Human Genetics, MRC IGMM, University of Edinburgh, Edinburgh, Scotland, UK; 8 Centre for Genomics and Experimental Medicine, Institute of Genetics and Molecular Medicine, University of Edinburgh, Western General Hospital, Edinburgh, UK; 9 Department of Psychology, University of Edinburgh, Edinburgh, UK

## Abstract

Cognitive impairment is common among individuals diagnosed with autism spectrum disorder (ASD) and attention-deficit hyperactivity disorder (ADHD). It has been suggested that some aspects of intelligence are preserved or even superior in people with ASD compared with controls, but consistent evidence is lacking. Few studies have examined the genetic overlap between cognitive ability and ASD/ADHD. The aim of this study was to examine the polygenic overlap between ASD/ADHD and cognitive ability in individuals from the general population. Polygenic risk for ADHD and ASD was calculated from genome-wide association studies of ASD and ADHD conducted by the Psychiatric Genetics Consortium. Risk scores were created in three independent cohorts: Generation Scotland Scottish Family Health Study (GS:SFHS) (*n*=9863), the Lothian Birth Cohorts 1936 and 1921 (*n*=1522), and the Brisbane Adolescent Twin Sample (BATS) (*n*=921). We report that polygenic risk for ASD is positively correlated with general cognitive ability (beta=0.07, *P*=6 × 10^−7^, *r*^2^=0.003), logical memory and verbal intelligence in GS:SFHS. This was replicated in BATS as a positive association with full-scale intelligent quotient (IQ) (beta=0.07, *P*=0.03, *r*^2^=0.005). We did not find consistent evidence that polygenic risk for ADHD was associated with cognitive function; however, a negative correlation with IQ at age 11 years (beta=−0.08, *Z*=−3.3, *P*=0.001) was observed in the Lothian Birth Cohorts. These findings are in individuals from the general population, suggesting that the relationship between genetic risk for ASD and intelligence is partly independent of clinical state. These data suggest that common genetic variation relevant for ASD influences general cognitive ability.

## Introduction

Autism spectrum disorder (ASD) and attention-deficit hyperactivity disorder (ADHD) are pervasive neurodevelopmental disorders that manifest during childhood. These disorders are highly heritable,^1, 2, 3, 4^ and recent genome-wide association studies (GWAS) have found that a portion of this heritability is attributable to common genetic variants.^5^ Cognitive difficulties are common in individuals with ADHD or ASD. Children with ADHD have been found to have a 7–12 point lower average full-scale intelligent quotient (FIQ) compared with controls.^6, 7^ Furthermore, individuals with ADHD demonstrate reduced working memory capacity and poorer processing speed and reading comprehension.^8, 9, 10, 11^ Executive function deficits in children with ADHD have been found to persist into adulthood^12^ despite remission of ADHD symptoms^13, 14^ although other studies find that impairment persists only in individuals whose ADHD remains.^15^ The relationship between autism and intelligence is, however, more complex. The majority of autistic individuals are intellectually impaired^16^ although a few studies have shown areas of superior functioning compared with controls,^17^ particularly on non-verbal measures.^18, 19^

General cognitive ability (g) is a latent trait that can be extracted from performance across diverse tests of cognitive aptitude. General cognitive ability has heritability estimates of approximately 30% in young childhood^20^ increasing to 80% in adolescence.^21^ Like ADHD and ASD, differences in general cognitive ability are highly polygenic, with about half of the heritability captured by common genetic variants.^22, 23, 24^ Despite a strong phenotypic relationship between autism, ADHD and cognitive decrements, few studies have examined the genetic overlap between these traits. A study examining the genetic correlation between five psychiatric disorders did not find significant genetic overlap between ADHD and ASD attributable to common single-nucleotide polymorphisms (SNPs).^5^ Twin studies suggest that there are shared genetic effects across autism,^25, 26^ ADHD and cognitive ability^7, 27^ although these studies are confounded by the clinical state of the affected individuals. Poor cognitive ability may arise via the social and communicative impairments present in autistic or ADHD individuals or the pleiotropic effect of genetic risk variants on cognition. A recent study found that rare copy number variants that increase risk for autism are associated with cognitive differences in healthy controls. Control carriers of the 16p11.2 deletion were significantly impaired on measures of verbal intelligence, working memory and executive function.^28^ However, no study to our knowledge has assessed the relationship between common genetic risk for ASD or ADHD and cognitive function in the general population.

The genetic overlap between different heritable traits can be investigated using polygenic risk profiling. This method takes the SNP effect sizes from a reference study and calculates the genome-wide weighted sum of the alleles that an individual carries, which then serves as an index of the genetic load for a particular disorder. This method has the advantage of analysing genetic overlap between cognitive ability, ADHD and ASD, without the confounding effect of disease state.

The aim of this study was to test whether ADHD and ASD polygenic profile scores^29^ are associated with cognitive ability in a large population-based cohort. Generation Scotland: Scottish Family Health Study (GS:SFHS)^30^ provides genome-wide genotyping data and four measures of cognitive function for 9863 individuals, some of whom are in family groups. Two other samples: the combined Lothian birth cohorts 1921 and 1936 (LBC1921 and LBC1936) (1522 individuals combined)^31^ and the Brisbane Adolescent Twin Study (BATS)^32^ were used for replication. LBC1921 and LBC1936 provided measures of cognitive function in childhood and old age for the same individuals. BATS consisted of 921 individuals with cognitive ability measured in adolescence. We hypothesized that individuals with a greater burden of ADHD or ASD risk alleles would perform worse on tests of cognitive function, consistent with findings in affected individuals. We also sought to test whether individuals with a high genetic load for ASD would perform better on non-verbal cognitive measures, similar to autistic individuals.

## Methods

### Cohort description and cognitive testing

#### Generation Scotland: Scottish Family Health Study (GS:SFHS)

GS:SFHS is a family-based epidemiological cohort; the protocol for recruitment is described in detail in the Supplementary Materials and in previous publications.^30^ Briefly, genome-wide SNP data were ascertained for 9863 individuals (mean age=52.2 years, s.d.=13.64), 5788 females and 4075 males (6815 unrelated participants). Four tests of cognitive function were available: Mill Hill vocabulary scale junior and senior synonyms,^33^ verbal declarative memory (logical memory),^34^ the Wechsler digit symbol substitution task (digit symbol coding (DSC)),^34^ and verbal fluency. A measure of general cognitive ability was derived by entering all four cognitive tests into a principal components analysis (PCA) and extracting the first unrotated principal component,^35^ which explained 44% of the variance across these tests. Each test loaded moderately onto the component (0.48–0.54). Further details on cognitive testing are included in the Supplementary Materials and summarized in Supplementary Table S1.

#### Lothian Birth Cohort 1936 (LBC1936)

The 1936 Lothian Birth Cohort (LBC1936) participants used in the present study consisted of 1005 (496 males and 509 females) community-dwelling individuals mostly living in and around the City of Edinburgh, Scotland. Almost all of them completed the Moray House Test (MHT) during the Scottish Mental Health Survey of 1947 at age 11 years, which was re-administered at a follow-up assessment at age 70 years (Scottish Research Council, 1933).^36, 37^ At age 70 years in LBC1936, the National Adult Reading Test (NART)^38^ and tests of logical memory, digit span backwards, spatial span and verbal paired associates were administered from the Wechsler memory scale (WMS-III UK).^39^ Tests from the Wechsler adult intelligence scale (WAIS-III) were also administered at age 70 years: DSC, block design, letter number sequencing, and matrix reasoning.^34, 40^ Verbal fluency^41^ and lifetime change in IQ were also assessed. General cognitive ability was constructed in LBC1936 by performing PCA of block design, matrix reasoning, letter number sequencing, digit span backwards, symbol search and DSC and extracting the first principal component. This component explained 53% of the variance across these six tests, (loading range 0.37–0.43). The LBC1936 cognitive tests are described in greater detail in the Supplementary Methods and summarized in Supplementary Table S1.

#### Lothian Birth Cohort 1921 (LBC1921)

The 1921 Lothian birth cohort (LBC1921) contributed 517 participants to the present study, 302 females and 215 males who completed the MHT at age 11 years as part of the Scottish Mental Health Survey in 1932.^42, 43^ The MHT was completed again at age 79 years. Participants completed the MHT at mean age 11 years and then again at mean age 79 years.^31^ At age 79 years, tests of logical memory,^39^ verbal fluency^41^ and Raven's progressive matrices^33^ were administered. These three tests were entered into PCA to derive general cognitive ability, and the first principal component explained 52% of the variance across these tests (loadings 0.58–0.64), summarized in Supplementary Table S1. Change in IQ from age 11 to age 79 years was ascertained as described for LBC1936.

#### Brisbane Adolescent Twin Sample (BATS)

BATS consists of 4500 individuals (~1800 families) and targets twin adolescents and their siblings, with the majority recruited through primary and secondary schools in South East Queensland.^32^ The present study used 921 genotyped individuals randomly selected, 1 per family (53.2% females; mean age=16.7 years, s.d.=1.6). Measures of cognitive function included FIQ^44^ (*N*=902), verbal IQ (VIQ) (Subtests: Information, Vocabulary, Arithmetic; *N*=903) and performance IQ (Spatial, Object Assembly; *N*=902). Processing speed was obtained from the Digit Symbol Substitution task, a subtest of the Wechsler Adult Intelligence Scale (WAIS-R)^40^ (*N*=874). Reading ability was assessed using a contextualized version of the NART^45^ (*N*=813). The tests used to create FIQ are summarized in Supplementary Table S1.

### Genotyping

#### GS:SFHS

Details of DNA extraction are described elsewhere.^30^ Briefly, blood samples were collected using standard operating procedures and stored using a laboratory information management system at the Wellcome Trust Clinical Research Facility Genetics Core, Edinburgh, UK (www.wtcrf.ed.ac.uk). DNA samples were genotyped by the WT-CRF using the IlluminaHumanOmniExpressExome -8v1.0 BeadChip (San Diego, CA, USA) and Infinum chemistry.^46^ Genotypes were processed using the GenomeStudio Analysis software v2011.1 (Illumina).

#### Lothian birth cohorts

Samples were subject to genome-wide genotyping via the extraction of venous blood from participants and genotyping at the Wellcome Trust Clinical Research Facility, Edinburgh, UK (www.wtcrf.ed.ac.uk). Genotyping was performed using the Illumina 610-Quadv1 whole-genome SNP array (Illumina, San Diego, CA, USA). The sample collection, quality control and genotyping process is described in greater detail elsewhere.^47^

#### BATS

Genome-wide genotyping data was collected (from DNA extracted from venous blood) using the Illumina 610-Quadv1 whole-genome SNP array. Quality control and imputation was carried out using the ENIGMA (Enhancing Neuro Imaging Genetics through Meta-Analysis) protocols.^48^ Imputation was carried out to the 1000G phase1 integrated reference panel (April 2012, NCBI build 37) using MiniMac, a computationally efficient implementation of the MaCH algorithm.^49^

### Polygenic profiling

**Figure 1:**
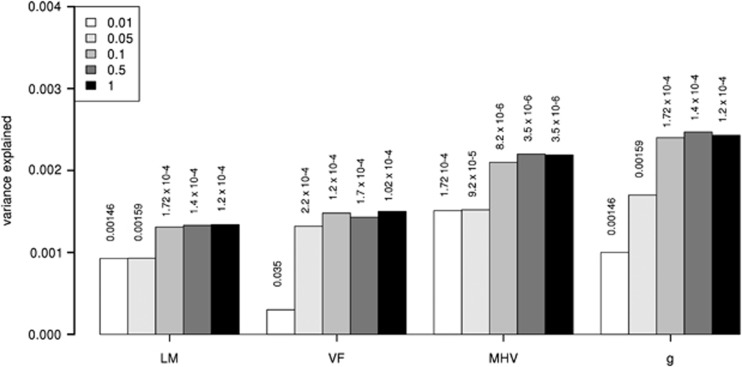
Proportion of variance explained in cognitive test performance explained by autism spectrum disorder polygenic risk score derived using five different *P*-value thresholds in the Generation Scotland: Scottish Family Health Study (GS:SFHS). Only tests significantly associated with polygenic risk score are presented. LM, logical memory; MHV, Mill Hill vocabulary; VF, verbal fluency.

The method to create polygenic risk scores has been previously described^50^ and is implemented in PLINK.^51^ Further detail is included in the Supplementary Methods. Summary statistics from the Psychiatric Genetics Consortium (PGC) cross-disorder GWAS, consisting of 4788 ASD trio cases, 4788 trio ASD pseudo controls, 161 ASD cases, 526 ASD controls and 1947 ADHD trio cases, 1947 trio ADHD pseudo controls, 840 ADHD cases and 688 ADHD controls,^5^ were used as the discovery set to create polygenic risk scores for ASD and ADHD. The PGC GWAS of ADHD and ASD explained 28% and 17% of the variance in liability to those disorders, respectively.^5, 29^ Any SNPs genotyped or imputed in our cohorts were used to create the polygenic risk scores, not just those showing statistically significant associations in the original GWAS. Five SNP set scores were generated, using *P*-value threshold cutoffs of 0.01, 0.05, 0.1, 0.5 and 1 from the original GWAS; however, as the SNP-set derived from a *P*-value threshold <0.5 explained the greatest amount of variance in cognitive function (Figure 1), only these results are presented throughout. Profile scores were generated using raw genotype data for GS:SFHS and the LBC. Polygenic profiling for the BATS cohort was performed on data imputed to the 1000 genomes data set using the ENIGMA2 protocol described elsewhere.^48^ For comparability of results, we calculated profile scores for GS:SFHS and the LBC using imputed genotypes and found the results to be largely consistent (see Supplementary Materials for details).

### Statistical analyses

#### Generation of Scotland cohort

Mixed linear model analyses were implemented in ASReml-R (www.vsni.co.uk/software/asreml), with cognitive test scores as the dependent variable and age, sex, the first four MDS components and polygenic risk score as fixed effects. Family structure was fitted as a random effect by creating the inverse of a relationship matrix using pedigree kinship information. *P*-values for fixed effects were calculated using Wald's conditional F-test. In order to calculate the variance in cognitive test scores explained by polygene score, the change in the sum of residual variance and the additive genetic variance after removing the polygenic risk score from the model and then dividing this by the sum of residual variance and the additive genetic variance. There is a substantial male preponderance in ASD and ADHD, and therefore the interaction between sex and each polygenic risk score was also analysed by fitting an interaction term in each model (Supplementary Table S6).

#### LBC cohorts

Linear regression analyses were implemented in the R statistical software package (http:www.r-project.org/), using models that adjusted for age, sex and the first four MDS components for population stratification. The cognitive phenotypes were assigned as the dependent variable with polygenic score for autism or ADHD as the independent variable. Results from the LBC1936 and LBC1921 analyses were combined together and a fixed-effect meta-analysis performed to increase power. Fixed-effect meta-analyses were carried out using the ‘meta' package implemented in R.

#### BATS

Linear regression analyses were carried out as in the LBC cohorts, implemented in STATA (version 11, Statacorp, College Station, TX, USA).

## Results

### Relationship of ASD polygenic score to cognition

**Figure 2:**
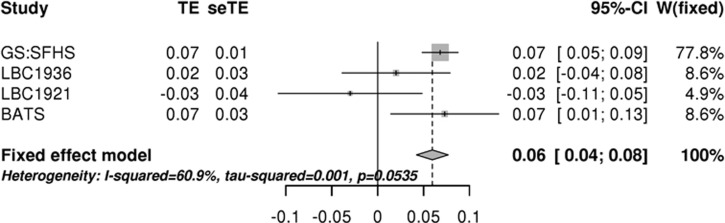
Forest plot showing effect size of polygenic risk for autism on general cognitive ability in the Generation Scotland Scottish Family Health Study (GS:SFHS), Lothian Birth Cohort (LBC; LBC1936 and LBC1921) and full-scale IQ in Brisbane Adolescent Twin Sample (BATS). CI, confidence interval; seTE, standard errors; TE, treatment effect (standardized regression coefficients); *W*(fixed), weight of individual studies in fixed-effect meta-analysis.

Polygenic risk for ASD was positively associated with general cognitive ability in GS:SFHS (beta=0.07, *P*=6 × 10^−7^). Polygenic load for ASD in GS:SFHS was also associated with three of the individual tests, delayed and immediate logical memory combined (beta=0.04, *P*=1 × 10^−4^), performance on the Mill Hill vocabulary test (beta=0.05, *P*=3 × 10^−6^) and verbal fluency (beta=0.04, *P*=2 × 10^−4^) (Table 1). These *P*-values all remain significant after Bonferonni correction. Greater polygenic risk for ASD was associated with better cognitive function across all four of these measures in this large adult population. The effect sizes are small. ASD polygenic scores explain <0.5% of the variance in cognitive test scores in all cases (Figure 1). A positive relationship between ASD polygenic risk scores and cognitive ability was also found in BATS with FIQ (beta=0.07, *P*=0.029) and VIQ (beta=0.07, *P*=0.029) (Table 2). A fixed-effect meta-analysis of general cognitive ability in GS:SFHS and LBC and FIQ in BATS found a significant positive association of ASD risk with cognitive ability (beta=0.06, *Z*=6.75, *P*<0.0001; Figure 2). In the LBC cohorts, no significant associations with ASD polygenic risk were found in the combined meta-analyses (Table 3). Additional cognitive tests derived from the WMS-III and WAIS-III were also tested for association with autism polygenic risk in LBC1936; however, no significant associations were found (Supplementary Table S2).

### Relationship of ADHD polygenic score to cognition

No significant associations between polygenic risk for ADHD and cognitive ability were found in GS:SFHS (Table 1). No significant associations between polygenic risk for ADHD and cognitive ability were found in BATS (Table 2). Polygenic risk for ADHD was associated with age 11 IQ in the LBC1936 cohort; however, in contrast to ASD, greater polygenic load was negatively correlated with IQ (beta=−0.09, *P*=0.004). This result was not replicated in LBC1921. A meta-analysis of the LBC1921 and LBC1936 revealed a significant association with age 11 IQ (beta=−0.08, *Z*=−3.3, *P*=0.001), and this *P*-value remains significant after Bonferonni correction for multiple testing. As previous studies have found significant genetic covariance between ADHD and depression,^5^ statistical analyses were also carried out on the GS:SFHS control individuals (*n*=7667) after removing those with a lifetime diagnosis of depression. A negative association between ADHD polygenic risk scores and DSC scores in GS:SFHS was significant when depressed individuals were removed from the analysis (beta=−0.02, *P*=0.03) (Supplementary Table S4).

## Discussion

This study found a positive association between common genetic risk variants for ASD and general cognitive ability in individuals drawn from the general population. ASD polygenic risk scores were also positively correlated with measures of verbal fluency, logical memory and vocabulary. In contrast, genetic risk for ADHD was negatively correlated with age 11 IQ in members of the combined Lothian Birth Cohorts, although this finding was not confirmed in any of the other cohorts. The degree of genetic overlap between ASD and ADHD attributable to common genetic variation has previously been shown to be non-significant,^5^ and therefore a differential effect of ASD and ADHD polygenic risk scores is plausible. These results provide evidence that common genetic variation associated with ASD confers better general cognitive ability in a non-clinical population.

The rate of intellectual disability in autistic individuals is estimated to be 70%^16^ although this is lower when broader ASDs are included.^52^ A recent study examined the impact of copy number variants associated with neuropsychiatric disease on cognition in healthy controls.^28^ A rare chromosome 16p11.2 deletion, which is a highly penetrant genetic risk factor in autism,^53^ was found to significantly impair cognitive function in healthy control carriers. In contrast, our results suggest that common genetic risk for autism correlates positively with cognitive function. The relationship between autism and intelligence may therefore be complicated by the existence of distinct classes of genetic risk. Autism has the highest twin heritability of all psychiatric disorders,^54, 55^ yet only 17% of this variance is attributable to common variants.^5^ This discrepancy is most likely owing to rare and *de novo* mutations, which are increasingly recognized as an important source of genetic risk for autism^56^ and are negatively associated with cognition in healthy individuals.^28^ Individuals with high functioning autism often display an atypical Wechsler intelligence profile, with strengths on matrix reasoning and block design and weaknesses on DSC and symbol search subtests.^57, 58, 59, 60^ We did not find any evidence that genetic risk for autism in non-clinical individuals was consistently associated with strengths or weaknesses in any of these domains. It is notable, however, that in the GS:SFHS, DSC was the only test of cognition where individuals with high polygenic profiles scores for autism did not perform better.

Individuals with ADHD typically have poorer cognitive abilities than their age-matched counterparts. Executive function deficits are found to persist into adulthood^8^ despite remission of ADHD symptoms^13, 14^ although other studies find impairments only in individuals whose ADHD remains.^15^ Symptoms of ADHD, such as hyperactivity and inattention, associate with scholastic impairment in children drawn from a population-based sample.^61^ Furthermore, polygenic risk for ADHD associates positively with hyperactivity and impulsivity in the general population.^62^ We provide suggestive evidence that polygenic risk for ADHD associates with ADHD-like traits in the general population by showing a negative relationship between age 11 IQ and ADHD genetic risk in the LBC. We also demonstrate a weak negative association with DSC in GS:SFHS when individuals with a lifetime diagnosis of depression were removed from the sample. Taken together, these data suggest that ADHD may lie on a continuous distribution of traits present in the general population; however, the association of ADHD polygenic risk and cognitive impairment should be interpreted cautiously and warrants further replication, as consistent evidence for this was not found across all cohorts in this study. Sex-specific analyses revealed a significant interaction between sex and ASD polygenic risk score in the context of DSC in GS:SFHS, indicating that genetic risk for ASD in females is positively correlated with performance on DSC, in contrast to males. There is a notable sex bias in ADHD and ASD prevalence, with roughly 2.5 males affected for every female for ADHD^63^ and 4 males for every female affected in ASD.^64^ Interestingly, these data show that the effects of ASD genetic risk on DSC performance are more pronounced in females from the general population.

There are a number of limitations to this study that should be noted. Consistent replication was not achieved across studies. Polygenic risk for autism, although robustly associated with cognition in GS:SFHS, only replicated in BATS. The failure to consistently replicate the finding in GS:SFHS may be due to the smaller sample size of the LBC cohorts. Furthermore, the LBCs comprise older participants and measuring general cognitive ability in this age group may have affected the results. Similarly, ADHD was found to associate with age 11 IQ in the LBC cohort, but this did not replicate in BATS or GS:SFHS, although the relationship between cognitive function and ADHD risk was negative in 4/5 tests in GS:SFHS. Furthermore, many of the significant *P*-values reported in this study are modest and do not survive correction for multiple testing. The lack of replication may have arisen from the unavoidable but significant heterogeneity among cognitive tests across the three cohorts. A lack of power to detect association in the smaller cohorts may also have contributed to the lack of replication. This is not unexpected when considering the amount of variance in cognitive function we were able to explain in GS:SFHS: <0.5% in all cases. This is consistent with other studies that have employed polygenic risk scoring to assess genetic overlap between disorders or across cohorts. According to recent estimates, 32% of the genetic liability for schizophrenia (SCZ) can be explained by common genetic variation; however, using polygenic risk scores to predict SCZ status in an independent cohort, only ~7% of the variance in liability could be explained.^65^ The genetic variance explained by common SNPs increases as a function of sample size. As the PGC GWAS of ASD and ADHD were considerably smaller than the SCZ GWAS (*N* for autism ~6700, ADHD ~16 000, SCZ ~150 000), the degree of polygenic overlap between autism, ADHD and cognition will be an underestimate.

The current study suggests that genetic risk for autism is positively correlated with cognitive function in non-clinical cohorts and is likely to be independent of ASD pathology. The genetic basis of ADHD and autism are poorly understood; however, understanding the significant and opposing directions of genetic covariance between these traits and cognitive function will provide opportunities to investigate their biological origins.

## Figures and Tables

**Table 1:** Polygenic risk for ASD/ADHD generated from SNPs with *P*-value cutoff threshold of 0.5 and tests of cognitive function in the Generation Scotland (*n*=9863) cohort, using mixed linear models implemented in ASReml-R, controlling for age and sex

*Risk score*	*Test*	*Solution*	*S.e*	Z-*ratio*	*Var*	P*-value*
ASD	DSC	0.011	0.01	1.182	2 × 10^−5^	0.237
	Logical Memory	0.039	0.01	3.814	0.001	**0.0001**
	MHV	0.047	0.01	4.64	0.002	**0.000003**
	Verbal Fluency	0.040	0.01	3.757	0.001	**0.0002**
	g	0.068	0.01	4.980	0.003	**6 × 10^−7^**
ADHD	DSC	−0.015	0.01	−1.702	0.0002	0.089
	Logical Memory	−0.009	0.01	−0.921	3 × 10^−7^	0.357
	MHV	−0.014	0.01	−1.397	5 × 10^−5^	0.162
	Verbal Fluency	0.002	0.01	0.145	0	0.884
	g	−0.010	0.01	−0.760	0	0.45

Abbreviations: ADHD, attention-deficit hyperactivity disorder; ASD, autism spectrum disorder; DSC, digit symbol coding; g, general cognitive ability; MHV, Mill Hill vocabulary; SNP, single-nucleotide polymorphism. *P*-value is derived from Wald Conditional F-test. Var, proportion of variance in test explained by polygene score. Bold values signify *P*≤0.05.

**Table 2:** Polygenic risk for ASD/ADHD in BATS sample (*n*=921) generated from SNPs with *P*-value cutoff threshold of 0.5 and the relationship with cognitive ability, controlling for age and sex

*Risk score*	*Test*	*Beta*	*S.e*	t*-value*	r^*2*^	P*-value*
ASD	FIQ	0.073	0.03	2.19	0.005	**0.029**
	PIQ	0.058	0.03	1.73	0.0028	0.085
	VIQ	0.073	0.03	2.19	0.005	**0.029**
	NART	0.023	0.04	0.65	0.0009	0.518
	DSC	0.059	0.03	1.87	0.001	0.062
ADHD	FIQ	0.042	0.03	1.29	0.002	0.199
	PIQ	0.057	0.03	1.71	0.0028	0.087
	VIQ	0.013	0.03	0.38	0.0003	0.702
	NART	0.018	0.04	0.52	0.0007	0.606
	DSC	0.017	0.03	0.56	0	0.579

Abbreviations: ADHD, attention-deficit hyperactivity disorder; ASD, autism spectrum disorder; BATS, Brisbane Adolescent Twin Sample; DSC, digit symbol coding; FIQ, full-scale intelligent quotient; NART, National Adult Reading Test; PIQ, performance IQ; SNP, single-nucleotide polymorphism; VIQ, verbal IQ. Bold values signify *P*≤0.05.

**Table 3:** Polygenic risk for ASD/ADHD generated from SNPs with *P*-value cutoff threshold of 0.5 and cognitive ability in LBC1936 (*n*=1005), LBC1921 (*n*=517) and combined fixed-effect meta-analysis (*n*=1522)

*Cognitive measure*	*Risk score*	*1936*	P*-value*	*1921*	P*-value*	*Meta-analysis*	P*-value*
ASD	Age 11 IQ	*β*=0.03, s.e.=0.03, *t*=0.88, *r*^2^=0.0008	0.38	*β*=−0.03, s.e.=0.04, *t*=−0.65, *r*^2^=0.0005	0.52	*β*=0.008, *Z*=0.35	0.73
	Age 70/79 IQ	*β*=0.04, s.e.=0.03, *t*=1.12, *r*^2^=0.001	0.26	*β*=−0.08, s.e.=0.04, *t*=−1.74, *r*^2^=0.009	0.08	*β*=−0.003, *Z*=−0.13	0.89
	CiIQ	*β*=0.02, s.e.=0.03, *t*=0.66, *r*^2^=0.0005	0.51	*β*=−0.11, s.e.=0.04, *t*=−2.34, *r*^2^=0.01	**0.02**	*β*=−0.03, *Z*=−1.12	0.26
	g	*β*=0.02, s.e.=0.03, *t*=0.6, *r*^2^=0.0003	0.55	*β*=−0.03, s.e.=0.04, *t*=−0.72, *r*^2^=0.001	0.48	*β*=0.002, *Z*=0.08	0.93
	Logical Memory	*β*=0.003, s.e.=0.03, *t*=0.09, *r*^2^=0	0.93	*β*=−0.037, s.e.=0.04, *t*=−0.82, *r*^2^=0.001	0.41	*β*=−0.01, *Z*=−0.48	0.63
	Verbal Fluency	*β*=0.05, s.e.=0.03, *t*=1.72, *r*^2^=0.003	0.09	*β*=−0.04, s.e.=0.04, *t*=−0.94, *r*^2^=0.002	0.35	*β*=0.02, *Z*=0.73	0.46
	NART	*β*=0.000, s.e.=0.03, *t*=0.006, *r*^2^=7 × 10^−5^	0.995	*β*=−0.03, s.e.=0.04, *t*=−0.64, *r*^2^=0.0008	0.52	*β*=−0.01, *Z*=−0.45	0.65
ADHD	Age 11 IQ	*β*=−0.09, s.e.=0.03, *t*=−2.85, *r*^2^=0.009	**0.004**	*β*=−0.06, s.e.=0.04, *t*=−1.36, *r*^2^=0.005	0.18	*β*=−0.08, *Z*=−3.3	**0.001**
	Age 70/79 IQ	*β*=−0.05, s.e.=0.03, *t*=−1.44, *r*^2^=0.002	0.15	*β*=−0.03, s.e.=0.04, *t*=−0.74, *r*^2^=0.003	0.46	*β*=−0.04, *Z*=−1.78	0.07
	CiIQ	*β*=0.03, s.e.=0.03, *t*=0.92, *r*^2^=0.0009	0.36	*β*=−0.01, s.e.=0.04, *t*=−0.29, *r*^2^=0.0002	0.78	*β*=0.016, *Z*=0.65	0.52
	g	*β*=−0.02, s.e.=0.03, *t*=−0.54, *r*^2^=0.0003	0.59	*β*=−0.06, s.e.=0.04, *t*=−1.31, *r*^2^=0.003	0.19	*β*=0.03, *Z*=1.43	0.15
	Logical Memory	*β*=−0.049, s.e.=0.03, *t*=−1.6, *r*^2^=0.002	0.11	*β*=−0.002, s.e.=0.04, *t*=−0.04, *r*^2^=0	0.97	*β*=−0.03, *Z*=−1.36	**0.017**
	Verbal Fluency	*β*=0.02, s.e.=0.03, *t*=0.76, *r*^2^=0.0006	0.45	*β*=−0.07, s.e.=0.04, *t*=−1.65, *r*^2^=0.005	0.10	*β*=−0.01, *Z*=−0.52	0.61
	NART	*β*=−0.07, s.e.=0.03, *t*=−2.457, *r*^2^=0.006	**0.01**	*β*=0.000, s.e.=0.04, *t*=−0.01, *r*^2^=0	0.99	*β*=−0.04, *Z*=−1.86	0.06

Abbreviations: ADHD, attention-deficit hyperactivity disorder; ASD, autism spectrum disorder; g, general cognitive ability; IQ, intelligent quotient; LBC, Lothian Birth Cohort; NART, National Adult Reading Test; SNP, single-nucleotide polymorphism. Bold values signify ≤0.05.
