# Supplementary material for: Common polygenic risk for autism spectrum disorder (ASD) is associated with cognitive ability in the general population
Source: Mol Psychiatry. 2015 Mar 10;21(3):419–25. doi: 10.1038/mp.2015.12 (PMC4759203; doi:10.1038/mp.2015.12)
Supplement: Supplementary Methods [file mp201512x1.doc]

**This document contains supplemental material for the manuscript by Clarke et al “Common polygenic risk for Autism Spectrum Disorders (ASD) is associated with cognitive ability in the general population”.**

**Supplemental Methods**

**Cohort Descriptions and cognitive testing**

**GS:SFHS:** GS:SFHS consists of 23,960 individuals recruited at random from general medical practices across Scotland. Eligibility criteria specified that participants were over 18 years of age and had one first-degree relative also willing to participate. Genome-wide SNP datawere ascertained for 10,000 individuals, and after quality control, genotype data were available for 9863 participants, which are the participants used in this study. 17% of the genotyped individuals had a diagnosis of single or recurrent episode depression. Verbal declarative memory was assessed using the sum of scores from the immediate and delayed recall section of one story of the Wechsler logical memory test ^1^. Verbal fluency, a test of executive function, was assessed using the letter-based verbal fluency test (letters C, F and L, for one minute each)^2^. Processing speed was measured using the Wechsler digit symbol substitution task (digit symbol coding) ^1^. Performance on digit symbol coding is partly attributed to sustained attention ^3^.

**LBC1936:** Participants completed the Moray House Test (MHT) (Scottish Council for Research in Education, 1933) at mean age 11^4, 5^. The MHT is a paper-and-pencil test with a time limit of 45 minutes which consists of primarily verbal reasoning items. Moray House Test scores were converted into an IQ-type scale, with a mean of 100 and a standard deviation of 15 as reported elsewhere ^6^. Logical memory was assessed using the sum of scores from the immediate and delayed recall sections. was assessed using the letter-based verbal fluency test using the letters C, F and L for one minute each. Lifetime change in IQ was analysed using linear regression, in which the residual value represents the deviation in IQ at aged 70 based on that predicted by age 11 IQ scores. This gives an estimate of cognitive change with a negative value representing a decline in function relative to other individuals in the cohort.

**BATS:** BATS individuals have participated in studies assessing traits such as melanocytic naevi (moles), cognitive function, brain structure and function, and mental health. The oldest participants, recruited in 1992, are now in their early 30’s. Over 2,700 have had cognitive function assessed, in most cases, at 16 years of age. Written informed consent was obtained from all participants and from a parent or guardian for those aged under 18 years. The study was approved by the QIMR Berghofer Human Research Ethics Committee. Full scale IQ (FIQ) ^7^was ascertained from five subtests of the Multi-dimensional Aptitude Battery (MAB) which also provided measures of verbal IQ (VIQ) and performance IQ.

**Polygenic profiling**

SNPs were excluded if they had a minor allele frequency (MAF) < 5%, deviated significantly from Hardy-Weinberg equilibrium (p<0.001) in the total sample of founder individuals, or had a call rate <98%. Individuals that had an overall SNP call rate of <95% were excluded from further analysis. Multi-dimensional scaling (MDS) components were created in the software package PLINK ^8^ to control for population stratification in downstream analysis. Briefly, strand-ambiguous SNPs were removed from the genotype data and clump based LD pruning (r^2^ 0.25, 300kb window) was performed to create a SNP-set in linkage equilibrium. Profile scores were generated by taking the sum of the PGC reference alleles weighted by the logarithm of odds ratio and divided by the number of SNPs used in the SNP set.

**Imputation protocols**

**GS:SFHS Imputation Protocol:** Genotype data was pre-phased using the SHAPEIT v2 software ^9^, and IMPUTE2 imputation software ^10^was used to impute genotypes to the 1000 genomes (1KG) reference panel (Phase I integrated release v3, April 2012). Indel call rate thresholds were set at 97%, SNP call rates at 98% and a MAF cut-off of 0.01 and a HWE p-value cut-off of 1 x 10^-6^ were used.

**Lothian Birth Cohort Imputation Protocol:** Imputation was performed using Minimac software ^10^, to the 1KG reference panel (Phase I integrated release v3, April 2012) according to the protocol specified in the 1000 Genomes Imputation Cookbook (http://genome.sph.umich.edu/wiki/Minimac:_1000_Genomes_Imputation_Cookbook).

**Polygenic risk profile calculation on imputed data:** Polygenic profiling in the BATS sample was carried out on imputed data using an r2 imputation quality threshold of 0.3 to filter out SNPs. SNPs were excluded if they had a minor allele frequency (MAF) < 2%, deviated significantly from Hardy-Weinberg equilibrium (p<0.000001) in the total sample of founder individuals, or had a call rate <99%. Individuals were excluded from further analysis that had an overall SNP call rate of <98%. Briefly, strand-ambiguous SNPs were removed from the genotype data and clump based LD pruning (r^2^ 0.2, 300kb window) was performed to create a SNP-set in linkage equilibrium. All SNPs which were associated with ADHD or autism in the PGC GWAS with a p<0.5 were used to create polygene scores, which were obtained by taking the sum of the reference alleles weighted by the logarithm of odds ratio and divided by the number of SNPs used in the SNP set. For consistency, polygenic profile scores in LBC and GS:SFHS were also created using this method, and the results reported in supplemental table two and four. The correlation between imputed polygenic risk scores and polygenic risk scores from raw genotype data was 0.82 in GS:SFHS and 0.78-0.81 in LBC1921 and LBC1936.

**Supplemental results**

**Sex * polygenic risk score interaction and the relationship to cognition**

The interaction between sex and polygenic risk and the impact on cognitive function was explored in the GS:SFHS cohort as this was the largest cohort available for these analyses. A significant interaction between sex and autism polygenic risk was observed when DSC was the dependent variable (beta=-0.04, p=0.026). Females with a high polygenic risk for autism performed better on DSC tests; however, this was not seen in males (Supplemental Table Five).

**References**

1. Wechsler D. WAIS-III UK Administration and Scoring Manual. *London* 1998; Psychological Corporation.

2. Lezak MD. Neuropsychological Assessment. *New York* 1995; Oxford University Press.

3. Schear JM, Sato SD. Effects of visual acuity and visual motor speed and dexterity on cognitive test performance. *Arch Clin Neuropsychol* 1989; 4(1): 25-32.

4. Deary IJ, Gow AJ, Pattie A, Starr JM. Cohort profile: the Lothian Birth Cohorts of 1921 and 1936. *Int J Epidemiol* 2011; 41(6): 1576-1584.

5. Deary IJ, Gow AJ, Taylor MD, Corley J, Brett C, Wilson V *et al.* The Lothian Birth Cohort 1936: a study to examine influences on cognitive ageing from age 11 to age 70 and beyond. *BMC Geriatr* 2007; 7: 28.

6. Gow AJ, Corley J, Starr JM, Deary IJ. Reverse causation in activity-cognitive ability associations: the Lothian Birth Cohort 1936. *Psychol Aging* 2013; 27(1): 250-255.

7. Jackson DN. Multidimensional Aptitude Battery II. *Port Huron, MI* 1998; Sigma Assessment Systems.

8. Purcell S, Neale B, Todd-Brown K, Thomas L, Ferreira MA, Bender D *et al.* PLINK: a tool set for whole-genome association and population-based linkage analyses. *Am J Hum Genet* 2007; 81(3): 559-575.

9. Delaneau O, Zagury JF, Marchini J. Improved whole-chromosome phasing for disease and population genetic studies. *Nat Methods* 2013; 10(1): 5-6.

10. Howie B, Fuchsberger C, Stephens M, Marchini J, Abecasis GR. Fast and accurate genotype imputation in genome-wide association studies through pre-phasing. *Nat Genet* 2012; 44(8): 955-959.

**Supplemental Tables**

|  | **Generation Scotland : the Scottish Family Health Study (GS:SFHS)**  **(Mean age 52.2)** | **Brisbane adolescent twin sample (BATS)**  **(Mean age 16.7)** | **Lothian Birth Cohort 1936 (age 70 testing)** | **Lothian Birth Cohort 1921 (age 79 testing)** |
| --- | --- | --- | --- | --- |
| **Components of ‘g’ or full scale IQ** | Digit Symbol Coding  Mill Hill Vocabulary  Verbal Fluency  Logical Memory | Multi-dimensional aptitude battery (MAB):  Verbal subtests (Information, vocabulary, arithmetic)  Performance subtests (spatial, object assembly) | Digit symbol Coding  Block design  Matrix reasoning  Letter number sequencing  Digit span backwards  Symbol search | Raven’s progressive matrices  Logical Memory  Verbal Fluency |

**Supplemental Table 1)** Showing which cognitive tests were used to create ‘g’ or full scale IQ (BATS) in each cohort and the ages at which testing was carried out.

| **Test** | **Risk Score** | **Beta** | **Standard Error** | **t value** | **r2** | **P-value** |
| --- | --- | --- | --- | --- | --- | --- |
| **Block Design** | ASD | 0.022 | 0.031 | 0.715 | 0.0001 | 0.475 |
|  | ADHD | 0.011 | 0.031 | 0.357 | 0.0001 | 0.721 |
| **Digit Span Backwards** | ASD | 0.004 | 0.032 | 0.141 | 0.0003 | 0.888 |
|  | ADHD | -0.039 | 0.032 | -1.227 | 0.002 | 0.220 |
| **Letter No. Seq** | ASD | 0.009 | 0.032 | 0.267 | 0 | 0.789 |
|  | ADHD | -0.039 | 0.032 | -1.233 | 0.0015 | 0.218 |
| **Spatial Span** | ASD | 0.031 | 0.031 | 0.997 | 0.0008 | 0.319 |
|  | ADHD | -0.042 | 0.032 | -1.323 | 0.002 | 0.186 |
| **SRT** | ASD | -0.026 | 0.032 | -0.814 | 0.0007 | 0.416 |
|  | ADHD | -0.006 | 0.032 | -0.179 | 0 | 0.858 |
| **Verbal Paired Associates** | ASD | 0.023 | 0.032 | 0.730 | 0.0004 | 0.465 |
|  | ADHD | -0.037 | 0.032 | -1.156 | 0.0013 | 0.248 |
| **Digit Symbol Coding** | ASD | -0.012 | 0.03 | -0.389 | 0.0001 | 0.698 |
|  | ADHD | 0.006 | 0.03 | 0.202 | 0 | 0.840 |
| **Matrix Reasoning** | ASD | 0.034 | 0.03 | 1.084 | 0.001 | 0.279 |
|  | ADHD | 0.018 | 0.03 | 0.584 | 0.0003 | 0.560 |
| **Symbol Search** | ASD | 0.056 | 0.03 | 1.825 | 0.003 | 0.068 |
|  | ADHD | -0.026 | 0.03 | -0.862 | 0.0007 | 0.389 |
| **Ravens Matrices** | ASD | 0.0004 | 0.04 | 0.09 | 0 | 0.92 |
|  | ADHD | -0.04 | 0.04 | -0.95 | 0.002 | 0.34 |

**Supplemental Table 2)** Polygenic scores for ASD/ADHD generated from SNPs with p-value cut-off threshold of 0.5, and cognitive ability tests at age 70 LBC1936, and with scores on Raven’s progressive matrices at age 79 in LBC1921. SRT = Simple Reaction Time

| **Test** | **Risk Score** | **Beta** | **Standard Error** | **Z – Ratio** | **Var** | **P-value** |
| --- | --- | --- | --- | --- | --- | --- |
| **Digit Symbol Coding** | ASD | 0.002 | 0.009 | 0.24 | 0 | 0.81 |
|  | ADHD | -0.011 | 0.009 | -1.19 | 6.3 x 10^-6^ | 0.23 |
| **Logical Memory** | ASD | 0.026 | 0.01 | 2.57 | 0.0005 | **0.01** |
|  | ADHD | -0.02 | 0.01 | -1.97 | 0.0003 | **0.05** |
| **Mill Hill Vocabulary** | ASD | 0.03 | 0.01 | 2.86 | 0.0007 | **0.004** |
|  | ADHD | -0.013 | 0.01 | -1.29 | 2.4 x 10^-4^ | 0.19 |
| **Verbal Fluency** | ASD | 0.029 | 0.01 | 2.81 | 0.0007 | **0.005** |
|  | ADHD | -0.001 | 0.01 | -0.14 | 0 | 0.89 |
| **g** | ASD | 0.04 | 0.01 | 3.20 | 0.002 | **0.001** |
|  | ADHD | -0.019 | 0.01 | -1.4 | 5.6 x 10^-5^ | 0.16 |

**Supplemental Table 3)** Polygenic risk for ASD/ADHD (calculated using imputed genotype data) generated from SNPs with p-value cut-off threshold of 0.5, and tests of cognitive function in Generation Scotland (n=9863) cohort using mixed linear models implemented in ASReml-R, controlling for age and sex. P-value derived from Wald Conditional F-test.Var = variance in test explained by polygene score (change in the sum of residual variance + additive genetic variance after dropping the polygene score from the model ).

| **Test** | **Risk Score** | **Beta** | **Standard Error** | **Z – Ratio** | **Var** | **P-value** |
| --- | --- | --- | --- | --- | --- | --- |
| **Digit Symbol Coding** | ASD | 0.01 | 0.01 | 1.05 | 1.26 x 10^-5^ | 0.29 |
|  | ADHD | -0.02 | 0.01 | -2.12 | 0.0003 | **0.034** |
| **Logical Memory** | ASD | 0.04 | 0.01 | 3.49 | 0.001 | **0.0005** |
|  | ADHD | -0.017 | 0.01 | -1.55 | 0.0002 | 0.12 |
| **Mill Hill Vocabulary** | ASD | 0.04 | 0.01 | 3.87 | 0.002 | **0.0001** |
|  | ADHD | -0.016 | 0.01 | -1.43 | 5.3 x 10^-5^ | 0.15 |
| **Verbal Fluency** | ASD | 0.036 | 0.01 | 2.99 | 0.001 | **0.002** |
|  | ADHD | 0.007 | 0.01 | 0.61 | 0 | 0.54 |

**Supplemental Table 4)** Polygenic risk for ASD/ADHD generated from SNPs with p-value cut-off threshold of 0.5 and tests of cognitive function in Generation Scotland in individuals without a lifetime diagnosis of depression (n=7667) using mixed linear models implemented in ASReml-R, controlling for age and sex. P-value derived from Wald Conditional F-test.Var = variance in test explained by polygene score (change in the sum of residual variance + additive genetic variance after dropping the polygene score from the model ).

| **Cognitive measure** | **Risk Score** | **1936** | **p-value** | **1921** | **p-value** | **Meta-analysis** | **p-value** |
| --- | --- | --- | --- | --- | --- | --- | --- |
| **Age 11 IQ** | **ASD** | β=0.02, S.E.=0.03, t=0.75, r^2^=0.0006 | 0.45 | β=0.01, S.E.=0.05, t=0.81, r^2^=0.0001 | 0.81 | β=0.02, Z=0.67 | 0.5 |
|  | **ADHD** | β=-0.06, S.E.=0.03, t=-1.81, r^2^=0.003 | **0.07** | β=-0.06, S.E.=0.05, t=-1.24, r^2^=0.003 | 0.22 | β=-0.06, Z=-2.33 | **0.02** |
| **Age 70 IQ – 1936**  **Age 79 IQ - 1921** | **ASD** | β=0.03, S.E.=0.03, t=0.8, r^2^=0.0006 | 0.42 | β=-0.04, S.E.=0.04, t=-0.86, r^2^=0.001 | 0.39 | β=0.005, Z=0.2 | 0.84 |
|  | **ADHD** | β=-0.02, S.E.=0.03, t=-0.51, r^2^=0.0003 | 0.61 | β=-0.05, S.E.=0.04, t=-1.03, r^2^=0.002 | 0.31 | β=-0.03, Z=-1.28 | 0.2 |
| **Change in IQ** | **ASD** | β=0.02, S.E.=0.03, t=0.75, r^2^=0.0006 | 0.45 | β=-0.07, S.E.=0.05, t=-1.42, r^2^=0.004 | 0.16 | β=-0.004, Z=-0.14 | 0.88 |
|  | **ADHD** | β=0.04, S.E.=0.03, t=1.18, r^2^=0.002 | 0.24 | β=-0.03, S.E.=0.05, t=-0.7, r^2^=0.001 | 0.49 | β=0.02, Z=0.83 | 0.4 |
| **g** | **ASD** | β=0.01, S.E.=0.03, t=-0.05, r^2^=0 | 0.96 | β=-0.03, S.E.=0.05, t=0.71, r^2^=0.001 | 0.48 | β=0, Z=-0.02 | 0.98 |
|  | **ADHD** | β=-0.03, S.E.=0.03, t=-0.86, r^2^=0.0007 | 0.39 | β=0.05, S.E.=0.04, t=1.14, r^2^=0.003 | 0.25 | β=-0.001, Z=-0.05 | 0.96 |
| **Logical Memory** | **ASD** | β=-0.004, S.E.=0.03, t=, r^2^=0 | 0.9 | β=-0.06, S.E.=0.04, t=-1.44, r^2^=0.004 | 0.15 | β=-0.03, Z=-1.007 | 0.31 |
|  | **ADHD** | β=-0.02, S.E.=0.03, t=-0.76, r^2^=0.0005 | 0.45 | β=0.02, S.E.=0.04, t=0.34, r^2^=0.0002 | 0.73 | β=-0.006, Z=-0.23 | 0.82 |
| **Verbal Fluency** | **ASD** | β=0.03, S.E.=0.03, t=0.98, r^2^=0.0009 | 0.33 | β=-0.04, S.E.=0.05, t=-0.79, r^2^=0.001 | 0.43 | β=0.01, Z=0.45 | 0.66 |
|  | **ADHD** | β=0.02, S.E.=0.03, t=0.56, r^2^=0.0003 | 0.58 | β=-0.08, S.E.=0.04, t=-1.8, r^2^=0.006 | 0.07 | β=-0.02, Z=-0.67 | 0.51 |
| **NART** | **ASD** | β=-0.04, S.E.=0.03, t=-1.27, r^2^=0.002 | 0.2 | β=-0.02, S.E.=0.04, t=-0.38, r^2^=0.003 | 0.70 | β=-0.03, Z=-1.37 | 0.17 |
|  | **ADHD** | β=-0.07, S.E.=0.03, t=-2.11, r^2^=0.004 | **0.04** | β=0.005, S.E.=0.04, t=0.12, r^2^=0 | 0.91 | β=-0.04, Z=-1.79 | **0.07** |

Supplemental Table 5) Polygenic risk for ASD/ADHD generated from SNPs with p-value cut-off threshold of 0.5, (calculated using imputed genotype data) and tests of cognitive function in LBC cohorts.

| **Interaction term** | **Cognitive test** | **Beta** | **Standard Error** | **Z ratio** | **P-value** |
| --- | --- | --- | --- | --- | --- |
| **ASD score * sex** | **Digit Symbol Coding** | -0.039 | 0.02 | -2.24 | **0.026** |
|  | **Logical Memory** | 0.034 | 0.02 | 1.74 | 0.08 |
|  | **Verbal Fluency** | 0.005 | 0.02 | 0.26 | 0.80 |
|  | **Mill Hill Vocabulary** | -0.007 | 0.02 | -0.34 | 0.74 |
|  | **g** | -0.01 | 0.03 | -0.37 | 0.71 |
| **ADHD score * sex** | **Digit Symbol Coding** | -0.033 | 0.017 | -1.91 | 0.057 |
|  | **Logical Memory** | -0.031 | 0.02 | -1.59 | 0.12 |
|  | **Verbal Fluency** | 0.011 | 0.02 | 0.57 | 0.57 |
|  | **Mill Hill Vocabulary** | -0.007 | 0.02 | -0.35 | 0.72 |
|  | **g** | -0.03 | 0.03 | -1.04 | 0.30 |

Supplemental Table 6) Interaction between polygenic risk score and sex and the relationship to cognitive ability in GS:SFHS.
